# Usefulness of the CDC/AAP and the EFP/AAP Criteria to Detect Subclinical Atherosclerosis in Subjects with Diabetes and Severe Periodontal Disease

**DOI:** 10.3390/diagnostics15070928

**Published:** 2025-04-04

**Authors:** Greicy C. Montenegro-González, Carlos Bea, F. Javier Ampudia-Blasco, Herminia González-Navarro, José T. Real, Maria Peñarrocha-Diago, Sergio Martínez-Hervás

**Affiliations:** 1 University of Valencia, 46010 Valencia, Spain; 2 Service of Internal Medicine, Hospital Clínico Universitario of Valencia, 46010 Valencia, Spain; carlos.bea@outlook.com; 3 INCLIVA Biomedical Research Institute, 46010 Valencia, Spain; ampudia_fra@gva.es (F.J.A.-B.); herminia.gonzalez@uv.es (H.G.-N.); jtreal@uv.es (J.T.R.); 4 Service of Endocrinology and Nutrition, Hospital Clínico Universitario of Valencia, 46010 Valencia, Spain; 5 Department of Medicine, University of Valencia, 46010 Valencia, Spain; 6 Department of Biochemistry and Molecular Biology, University of Valencia, 46010 Valencia, Spain; 7 CIBER de Diabetes y Enfermedades Metabólicas asociadas (CIBERDEM), Institute of Health Carlos III, Minister of Science, Innovation and Universities, 28029 Madrid, Spain; 8 Department of Stomatology, Faculty of Medicine and Dentistry, University of Valencia, 46010 Valencia, Spain; maria.penarrocha@uv.es

**Keywords:** CDC/AAP criteria, EFP/AAP criteria, periodontal disease, diabetes, atherosclerosis

## Abstract

**Background/Objectives**: Periodontitis is an inflammatory disease associated with many systemic disorders such as diabetes and cardiovascular disease. The aim was to evaluate the usefulness of the CDC/AAP and the EFP/AAP criteria to detect subclinical atherosclerosis in subjects with diabetes and severe periodontal disease. **Methods**: This was a cross-sectional study. Atheroma plaque was evaluated by high-resolution carotid and femoral ultrasonography. A dental examination protocol was implemented by a trained periodontist. A full-mouth periodontal clinical examination was carried out at six sites by automated computerized Florida Probe Periodontal Probing. Periodontal disease was defined by CDC/AAP and EFP/AAP criteria. **Results**: In total, 98 patients were included (60.2% women), of which 50% had diabetes. Subjects with diabetes showed a high prevalence of severe cases of periodontal disease. Both criteria were useful to detect the presence of atheroma plaque only in the presence of diabetes. However, the CDC/AAP criteria had higher correlation with atheroma plaques than EFP/AAP criteria (r = 0.522 vs. r = 0.369, *p* < 0.001). **Conclusions**: The CDC/AAP and the EFP/AAP criteria are a useful tool to identify subclinical atherosclerosis in subjects with severe periodontal disease and diabetes. These results show the potential role of the oral healthcare team in the dental office for the identification of subjects with diabetes at risk of developing cardiovascular disease.

## 1. Introduction

Periodontitis is one of the most prevalent diseases, affecting up to 50% of the worldwide population, and it is estimated that almost 20% of the global population are affected by severe forms [1,2]. It is characterized by a bacterially induced chronic inflammatory disease that ultimately can result in tooth loss [3,4]. However, periodontal disease is not just a localized disorder affecting oral health. Periodontitis has also been associated with many systemic disorders with common underlying pathological mechanisms. Several studies have suggested the existence of a bi-directional link between periodontal health, diabetes and cardiovascular disease, considering periodontal disease as a risk factor for both [4,5].

The link between diabetes and periodontal disease has been clearly established. Type 1 and type 2 diabetes are characterized by hyperglycemia and chronic inflammatory state contributing to micro and macrovascular complications [6,7]. Hyperglycemia also appears to change the systemic and gingival microvasculature, causing the periodontal tissues to become more inflammatory. Poor glycemic control and prolonged hyperglycemia have been implicated as risk factors for increased prevalence and severity of periodontal disease [8]. On the other hand, severe periodontitis seems to contribute to worse glycemic control. Different studies have suggested that periodontitis can play a role in the incidence of new cases of type 2 diabetes. Furthermore, effective periodontal therapy could reduce hyperglycemia in patients with poorly controlled diabetes [9,10].

Cardiovascular disease (CVD) is the leading cause of death in the world [11]. Current evidence supports the relationship between periodontal disease and CVD [12]. Both diabetes and periodontal disease are also a common global burden, associated with increased risk of atherosclerotic cardiovascular disease [13,14,15]. Subjects with diabetes present 2- to 4-fold higher mortality of cardiovascular origin [16]. Epidemiological studies have shown a positive association between periodontitis and coronary heart disease. Furthermore, the presence of viable invasive periodontal pathogens at the sites of atherosclerotic disease has been demonstrated [17]. Moreover, previous data suggest that there is an interaction between periodontal disease and diabetes, with both being associated with increased prevalence of subclinical and clinical atherosclerosis [18].

Therefore, early and accurate diagnosis of periodontal disease is crucial to initiate adequate therapeutic protocols to avoid irreversible tooth loss. Moreover, because periodontal health also impacts systemic health, treating periodontitis could possibly improve associated systemic diseases [4,19].

Different periodontitis case definitions have been proposed in recent years. The most widely used in epidemiological and clinical research has been the CDC/AAP (Centers for Disease Control/American Academy of Periodontology) case definition proposed in 2012 [20]. It has been considered by some authors as the gold standard method with which to compare [21]. In 2017, a new periodontal disease classification was adopted jointly by the European Federation of Periodontology (EFP) and the AAP (EFP/AAP) to facilitate implementation in general dental practice [22]. However, various studies have found differences in the prevalence and the severity of periodontal disease between both definitions [21,23,24,25].

Therefore, the aim of the present study was to compare the accuracy of the 2012 CDC/AAP and the 2017 EFP/AAP criteria to detect subclinical atherosclerosis in subjects with diabetes, which is a model of high risk for both periodontal disease and cardiovascular disease.

## 2. Materials and Methods

### 2.1. Study Design and Population

This cross-sectional study was reported according to STROBE guidelines [26]. The interventions were approved by the Ethical Committee of the Hospital Clinico Universitario of Valencia (reference: 2019/282) and performed according to the declaration of Helsinki.

We included 98 subjects (59 women and 39 men). The participating individuals were recruited through consecutive opportunistic sampling among those who attended the out-patient clinic of Endocrinology in the Hospital Clinico Universitario of Valencia during a period of 6 months, from January 2022 to June 2022.

The study included individuals aged ≥ 25 years with and without diabetes who had not received periodontal treatment in the six previous months and who had at least six teeth in the dental arch. The diagnosis of diabetes was considered according to the ADA criteria [27]: HbA1c ≥ 6.5%, fasting plasma glucose ≥ 126 mg/dL, 2 h plasma glucose ≥ 200 mg/dL or random glucose ≥ 200 mg/dL accompanied by classic hyperglycemic symptoms or hyperglycemic crises.

Exclusion criteria were the presence of a chronic inflammatory disease, previous cardiovascular disease, any inflammatory or infectious disease in the four weeks previous to the inclusion, periodontal treatment in the previous six months and less than six teeth in the dental arch.

To account for the effects of potential confounding factors, data were collected on gender, age, smoking status, presence of diabetes, anthropometric parameters, levels of fasting biochemical parameters, presence of atheroma plaque and presence of periodontal disease.

Diabetes, atheroma plaque and periodontal disease were defined as present or not.

The outcomes of the present study were the presence of periodontal disease and its severity and the presence of atherosclerosis.

The study protocol was approved by the Ethics Committee of the Hospital Clinico Universitario of Valencia (Ref 2019/282). All participants gave written informed consent before in inclusion in the study.

### 2.2. Clinical and Anthropometric Parameters

In the study protocol, the following clinical parameters were recorded using a standardized procedure: weight (kg), height (m), body mass index (BMI, kg/m^2^), waist circumference (midpoint between the edge lower rib and iliac crest, in cm), blood pressure and smoking habit. All these measurements were performed by the same investigator.

### 2.3. Laboratory Methods

After a 12 h fast, blood samples were drawn from an antecubital vein in tubes containing EDTA (Vacutainer) and were centrifuged within 4 h. Plasma was stored at 4 °C for a maximum of 3 days. Glucose, hbA1c, total cholesterol, triglycerides, HDL-c and Apo B were measured using standardized and validated methods as previously described. The LDL-c was calculated by Friedewald’s formula [28].

### 2.4. Carotid and Femoral Ultrasound

High-resolution carotid and femoral ultrasonography were performed with the 8 to 12 MHz transducer of a GE Logiq F6 ultrasound scanner (GE Healthcare, Chicago, IL, USA). Carotid examination was performed with the subjects in the supine position with the head turned 45° away from the side being explored. Four predetermined segments were evaluated on both sides: common carotid (1 cm proximal to the carotid bulb), carotid bulb (1–2 cm), and internal and external carotid (1 cm distal to the bifurcation). Evaluation was performed bilaterally in 3 different projections (right side: 90, 120 and 150°; left side: 210, 240 and 270°). Femoral examination was performed with the subjects in supine position. Two predetermined segments were evaluated on both sides: common femoral and superficial femoral. Atheroma plaque was considered in the presence of a focal thickening of more than 50% of the surrounding vessel wall or an IMT (intima–media thickness) greater than 1.5 mm that protruded into the adjacent lumen [29]. All examinations were performed by the same investigator (SM-H) trained in performing vascular ultrasounds and always following the identical protocol. The coefficient of variability was previously studied in 20 subjects and was 5.2% for the mean IMT of both common carotids.

### 2.5. Dental Examination Protocol

The dental examination protocol was developed at the University Dental Clinic of the University of Valencia. A full-mouth periodontal clinical examination was carried out at six sites (mesio-buccal, mid-buccal, disto-buccal, disto-lingual, mid-lingual, and mesio-lingual) per tooth excluding third molars, with standardized light and humidity conditions. A trained calibrated periodontist (GCM-G) conducted all the examinations, using magnifying loupes (Galilean HD HR ×2.5, 420 mm/16″; Akura Medical, Madrid, Spain) and automated computerized Florida Probe Periodontal Probing (Florida Probe Corp., Gainesvile, FL, USA).

The following parameters were recorded: number of teeth, gingival bleeding index (GBI), probing pocket depth (PPD), and clinical attachment level (CAL). PPD was measured from the free gingival margin to the bottom of the pocket/sulcus. CAL was defined as the distance from the cemento-enamel junction to the bottom of the pocket/sulcus.

### 2.6. Definition of Periodontal Disease

For the CDC/AAP case definition, a periodontitis case was considered as follows: mild periodontitis was defined as ≥2 interproximal sites with ≥3 mm CAL and ≥2 interproximal sites with ≥4 mm PPD that are not on the same tooth or ≥1 interproximal site with ≥5 mm PPD; moderate periodontitis was defined as ≥2 interproximal sites with ≥4 mm CAL, or ≥2 interproximal sites with ≥5 mm PPD, that are not on the same tooth; and severe periodontitis was defined as ≥2 interproximal sites, in different teeth, with ≥6 mm CAL and ≥1 interproximal site with ≥5 mm PPD. Gingivitis was diagnosed when the previously mentioned criteria were not met but had a GBI > 10% and PPD ≤ 3 mm. Finally, healthy periodontal cases were considered when none of the above criteria was present [20].

For the AAP/EFP case definition, a periodontitis case was considered when a patient has interproximal clinical CAL at ≥2 non-adjacent teeth or buccal or oral free faces CAL ≥ 3 mm with PPD ≥ 3 mm at ≥2 teeth; this CAL cannot be ascribed to non-periodontitis-related, for example gingival recession of traumatic origin, dental caries in the cervical area, CAL on second molar and associated with malposition or extraction of a third molar, an endodontic lesion or vertical root fracture [22].

The stage definition of the AAP/EFP was also considered. Stage I: highest interproximal clinical CAL of 1 to 2 mm; maximum PPD ≤ 4 mm; Stage II: highest interproximal clinical CAL of 3 to 4 mm, maximum PPD ≤ 5 mm; Stage III: highest interproximal clinical CAL ≥ 5 mm, PD ≥ 6 mm; and Stage IV: highest interproximal clinical CAL ≥ 5 mm, PD ≥ 6 mm but eight or more missing teeth by periodontal reasons. Stages III and IV were considered as severe periodontitis [22].

### 2.7. Risk of Bias

The possibility of bias exists. This is a cross-sectional study. Thus, causal inference is not possible. Only clinical parameters were considered; inflammatory markers of atherosclerosis and microbiologic factors were not addressed. Radiography to evaluate bone loss was not performed. Furthermore, the impact of systemic inflammation on both periodontitis and atherosclerosis cannot be excluded.

### 2.8. Statistical Analysis

Power analysis was performed to calculate the sample size using the GPower 3.1.9.7 programme [30]. The calculated sample size was 98 individuals (49 at each group) with given α (0.05), power (0.9) and effect size (0.6).

Data were analyzed using the Statistical Package for the Social Sciences (SPSS 28 for Windows; SPSS, Chicago, IL, USA). The results are expressed as mean ± standard deviation for quantitative variables and as percentages and/or total number for qualitative variables.

Normal distribution was evaluated for each variable. To compare normally distributed quantitative variables between groups, Student’s *t* test and the ANOVA test were used (2 or more variables, respectively). For non-normally distributed variables, the Mann–Whitney test and the Kruskal–Wallis test were used (2 or more variables, respectively). To correct confounding factors in some comparison studies, ANCOVA analysis was used. To compare the qualitative variables between groups, the Chi-square test was used, or the Fisher test when the number was less than 5. The bivariate correlations between variables were studied with the Pearson test for variables with a normal distribution and Spearman test for variables without normal distribution.

The accuracy of the AAP/EFP and CDC/AAP criteria to detect individuals with atheroma plaque was analyzed using ultrasonography exploration as the reference standard. In both cases, the sensitivity, specificity, positive predictive value (PPV), negative predictive value (NPV), and area under the receiver operating curve (ROC) were assessed.

To analyze the factors associated with the presence of atheromatous plaque, logistic regression analyses were performed. The *p*-values were two-tailed. Differences were considered statistically significant if the *p* value was less than 0.05.

## 3. Results

We included 98 patients (60.2% women) in this study, of which 50% had diabetes. The general characteristics of the subjects included are shown in Table 1. We found significant differences in the glucose metabolism parameters and in the lipid profile between both groups. All the subjects included in the study, independently of the presence of diabetes, had any degree of periodontal disease according to the 2017 EFP/AAP criteria, with most of them showing severe forms (68.4% had stages III and IV). On the contrary, according to the 2012 CDC/AAP criteria, only 15.3% of the patients were included in the severe group. Furthermore, patients with diabetes showed a higher frequency of periodontal disease, severe periodontal disease and prevalence of atheroma plaques compared to patients without diabetes.

The analysis of the accuracy of both periodontal disease criteria classifications to detect the presence of atheroma plaque in subjects with severe periodontal disease is shown in Table 2. In patients with diabetes, the CDC/AAP criteria were useful to identify subjects with atheroma plaque with good sensitivity (84.6%), although specificity was low. However, the EFP/AAP criteria showed higher specificity (84.6%) but lower sensitivity (67.6%). Moreover, the CDC/AAP criteria showed higher AUC ROC (0.833 vs. 0.749 for the EFP/AAP criteria). In subjects without diabetes, the results were similar, although the AUC ROC was lower.

We also explored the correlation between periodontal disease classifications and the variables analyzed (Table 3). Both criteria were significantly associated with age, smoking habit and the presence of atheroma plaque. The CDC/AAP criteria were also associated with glucose control. Although both criteria were significantly associated with the presence of atheroma plaque, the CDC/AAP criteria had higher correlation with atheroma plaque than the EFP/AAP criteria (r = 0.522 vs. r = 0.369, *p* < 0.001).

Finally, we also evaluated the possible association of different factors implicated in the development of atheroma plaque (Table 4). We found that CDC/AAP criteria were significantly associated with the presence of atheroma plaque only in the presence of diabetes. However, the EFP criteria did not reach a significant association.

## 4. Discussion

In this cross-sectional study in subjects with and without diabetes, we evaluated the concordance between the 2012 CDC/AAP and the 2017 EFP/AAP criteria to detect subjects with subclinical atherosclerosis. We confirmed a high prevalence of periodontal disease in patients with diabetes. In addition, our results also confirmed that severe periodontitis was associated with the presence of subclinical atherosclerosis. Although both criteria were useful to detect subjects with atheroma plaques, the CDC/AAP criteria were more accurate.

Diagnosing the most severe forms of periodontitis is of crucial importance to reduce the risk of tooth loss. Moreover, severe periodontitis has also been associated with systemic diseases such as diabetes and atherosclerosis [5]. We have found a high prevalence of periodontal disease. All the subjects included in the study, independently of the presence of diabetes, had some degree of periodontal disease according to the 2017 EFP/AAP criteria, with most of them showing severe forms (68.4% had stages III and IV). On the contrary, according to the 2012 CDC/AAP criteria, only 15.3% of the patients were included in the severe group, and one third did not have periodontal disease. The higher frequency of severe cases by the 2017 EFP/AAP classification is in agreement with other studies [20,23,31]. When we analyzed only the patients with diabetes, we found, as expected [32], a higher prevalence of periodontal disease compared to subjects without diabetes, showing more severe forms. Almost 75% of patients with diabetes were classified as stage III–IV by the EFP/AAP criteria. However, only 28.6% had severe disease according to the CDC/AAP criteria.

Our main objective was to evaluate whether periodontitis criteria were useful to detect subclinical atherosclerosis in subjects with diabetes. The association between diabetes, periodontal disease and atherosclerosis is bidirectional [4,33]. Diabetes is also a risk factor for both atherosclerosis and periodontal disease [34]. In fact, some authors have considered severe periodontal disease as the sixth major chronic complication of diabetes [35]. Cardiovascular disease is the principal cause of death in diabetes [6,7]. Evidence supports that periodontal disease is associated with increased risk of atherosclerosis and cardiovascular disease [36,37]. Different mechanisms have been involved [38]. Periodontitis causes both local and systemic inflammatory and immune responses, which play an important role in the pathogenesis of atherosclerosis [39]. Moreover, pathogenic bacteria from the oral cavity have been found in atherosclerotic lesions, showing viability when culturing the atheroma samples [14]. Some studies have even suggested that successful periodontal treatment could influence the progression of atherosclerotic cardiovascular disease [33]. The higher prevalence of atherosclerosis and periodontal disease in patients with diabetes could be explained by an earlier and more extensive systemic inflammatory response together with direct periodontal pathogen-induced endothelial damage [40,41,42]. Our results are consistent with other studies demonstrating a higher prevalence of carotid plaque in subjects with diabetes and severe periodontitis [43]. In the same line, Southerland et al. found that individuals with diabetes and severe periodontal disease had increased prevalence of subclinical and clinical atherosclerosis compared to those without periodontal disease or diabetes [18]. Furthermore, the risk of atherosclerotic plaque in hyperglycemic patients increases according to the severity of periodontal disease [44]. Thus, there is an interaction between severe periodontal disease and diabetes, with both being associated with atherosclerosis development.

Although some studies have evaluated the association between periodontal disease and atherosclerosis [45], there are no previous data concerning the utility of periodontitis classifications to detect subjects with subclinical atherosclerosis. Furthermore, there are scarce data about the relation between periodontal disease and the presence of atheroma plaque because previous studies have evaluated the carotid IMT. We have selected diabetes because it is a clinical model of high risk for developing atherosclerosis and periodontal disease [46]. We found that although both criteria were significantly correlated with atheroma plaque, there was a stronger association for the 2012 CDC/AAP criteria (r 0.522 vs. 0.369 *p* < 0.001 for CDC/AAP and EFP/AAP, respectively). Moreover, according to the CDC/AAP criteria, 80% of patients with severe periodontal disease had atheroma plaque but only 46.3% of patients in stage III-IV of the EFP/AAP classification had atheroma plaque. We also analyzed the sensitivity and specificity of both classifications to detect the presence of atheroma plaque when subjects were classified as severe forms. The CDC/AAP criteria showed higher sensitivity to detect atheroma plaque (84.6% vs. 67.6% for EFP/AAP) but lower specificity (58.8% vs. 84.6% for EFP/AAP). We also evaluated which the most important variables associated with the presence of atheroma plaque were among known cardiovascular risk factors and the presence of periodontal disease. When analyzing diabetes, the CDC/AAP criteria showed the strongest association with the presence of atheroma plaque (OR 18.868, *p* = 0.028). However, the EFP/AAP criteria had no significant association. Neither criteria showed significant association for detecting atheroma plaque in non-diabetic subjects.

Another important fact, based on our results and on previous studies, is the potential clinical implications of implementing periodontal disease screening as a predictive tool for cardiovascular risk in patients with diabetes. Integrating oral health into diabetes management is well-supported by the literature [47,48]. NICE recommends a care pathway that includes contributions from dental teams to identify individuals at high risk of type 2 diabetes [49]. Furthermore, the ADA’s Standards of Care in Diabetes, in the current 2025 recommendations, have added a new subsection, “dental care”, highlighting the importance of including dental health professionals in the diabetes care team and recommending that individuals with diabetes should be referred for a dental exam at least once per year [50]. Moreover, it would also be of great interest to screen subjects with diabetes and severe periodontitis concerning the risk of developing cardiovascular disease at the dental office. Diagnosing and treating early periodontal disease could potentially result in a delay in the development of atherosclerosis. However, this concept is still in early development and would require the implementation of protocols, as well as an evaluation of the usefulness of these actions.

A main strength of this preliminary analysis was that the same periodontist evaluated all the patients in a university setting using an automated probe (Florida probe) which applies constant probing force with precise electronic measurement contributing to the consistency of the measurements avoiding potential errors [51,52,53]. Furthermore, the use of magnifying loupes also contributed to the correct evaluation. Finally, we evaluated the presence of atheroma plaque (carotid and femoral) instead of IMT. Current cardiovascular risk guidelines consider that carotid or femoral plaque burden with ultrasound has been demonstrated to be predictive of cardiovascular events, comparable to coronary artery calcium (CAC), while the measurement of the carotid IMT is inferior to CAC score and carotid plaque detection [13,54].

However, the present study also has important limitations. First, we have included only a small sample size. Additionally, our data are relative only to a population at high risk of atherosclerosis and periodontitis, limiting the generalization of the present findings. In addition, the possibility of misclassification of periodontitis exists. On the one hand, we did not radiographically evaluate the bone loss. On the other hand, tooth loss may not be due to periodontitis. Furthermore, the impact of systemic inflammation on both periodontitis and atherosclerosis cannot be excluded. Finally, as this research was a cross-sectional study, the relationships found can only be used for the generation of hypotheses and cannot be interpreted as causal, making prospective studies necessary.

## 5. Conclusions

The CDC/AAP and the EFP/AAP criteria are a useful tool to identify subclinical atherosclerosis in subjects with severe periodontal disease and diabetes. This association may be of interest because identifying subjects at risk would contribute to intensifying the treatment of other cardiovascular risk factors to prevent the development of cardiovascular disease. Furthermore, preventing or treating periodontal disease could potentially contribute to reducing the progression of atherosclerosis and therefore the risk of developing major adverse cardiovascular events. Randomized clinical trials involving standardized periodontal interventions are necessary to evaluate whether periodontal treatment is beneficial to reduce cardiovascular disease risk. Finally, these results show the potential role of the oral healthcare team to identify subjects with diabetes at risk of developing cardiovascular disease in the dental office. This procedure should be evaluated and implemented in the dental office, calculating the cost-effectiveness of this screening and confirming its usefulness in larger populations.

## Figures and Tables

**Table 1:** Characteristics of the population according to the presence of diabetes.

Group	Complete Group (*n* = 98)	Non-Diabetes (*n* = 49)	Diabetes (*n* = 49)
Women/men *n*	59/39	37/12	22/27
Age (years old)	51.1 ± 14.8	48.2 ± 14.3	54.1 ± 14.8
BMI (kg/m^2^)	26.4 ± 4.7	26.2 ± 5.2	26.6 ± 4.4
Waist circumference (cm)	87.6 ± 15.1	86.5 ± 15.8	92.8 ± 12.5
SBP (mmHg)	126.2 ± 18.1	119.1 ± 15.8	131.2 ± 18.1 *
DBP (mmHg)	78.1 ± 9.6	76.7 ± 10.2	79.0 ± 9.2
Glucose (mg/dL)	114.4 ± 40.9	92.3 ± 10.4	136.5 ± 47.7 *
HbA1c (%)	6.2 ± 1.1	5.3 ± 0.3	7.1 ± 0.9 *
Total cholesterol (mg/dL)	171.9 ± 38.3	186.6 ± 29.4	156.9 ± 40.6 *
HDL-C (mg/dL)	60.0 ± 16.5	64.2 ± 11.6	55.9 ± 19.5 *
LDL-C (mg/dL)	104.2 ± 30.2	115.1 ± 24.9	93.4 ± 31.2 *
Triglycerides (mg/dL)	102.3 ± 88.4	81.4 ± 32.8	123.2 ± 117.6
Apo B (mg/dL)	89.4 ± 22.5	94.5 ± 20.2	84.1 ± 23.7 *
Diabetes	49 (50.0)	0 (0)	49 (100)
Smoking habit *n* (%)	10 (10.2)	3 (6.1)	7 (14.3)
Peridontal disease *n* (%) CDC/AAP criteria EFP/AAP criteria	64 (65.3) 98 (100)	27 (55.1) 49 (100)	37 (75.5) 49 (100)
Severe periodontal disease *n* (%) CDC/AAP criteria EFP/AAP criteria	15 (15.3) 67 (68.4)	1 (2.0) 31 (63.3)	14 (28.6) 36 (73.5)
Atheroma plaque *n* (%)	35 (35.7)	10 (20.0)	25 (51) *

Abbreviations: AAP, American Academy of Periodontology; Apo B, apolipoprotein B; BMI, body mass index; CDC, Center for Disease Control; DBP, diastolic blood pressure; EFP, European Federation of Periodontology; HbA1c, glycated hemoglobin; HDL-C, HDL cholesterol; LDL-C, LDL cholesterol; SBP, systolic blood pressure. Data are shown as mean ± standard deviation. * *p* < 0.05 between diabetes and non-diabetes.

**Table 2:** Diagnostic accuracy of both diagnostic criteria (CDC/AAP and EFP/AAP) to predict atheroma plaque in subjects with severe periodontal disease: A. subjects without diabetes; B. subjects with diabetes.

A.						
	CDC/AAP			EFP/AAP		
Atheroma Plaque	Yes	No	Total	Yes	No	Total
YES	1	9	10	8	2	10
NO	0	38	38	23	15	38
TOTAL	1	47	48	31	17	48
Sensitivity	66.7%			25.8%		
Specificity	80.8%			88.2%		
PPV	10%			80%		
NPV	97.9%			35.4%		
AUC ROC	0.637 (0.434–0.840)			0.607 (0.415–0.798)		
	*p* = 0.208			*p* = 0.459		
B.						
	**CDC/AAP**			**EFP/AAP**		
**Atheroma Plaque**	**Yes**	**No**	**Total**	**Yes**	**No**	**Total**
YES	20	2	22	11	11	22
NO	14	11	25	2	23	25
TOTAL	34	13	47	13	34	47
Sensitivity	84.6%			67.6%		
Specificity	58.8%			84.6%		
PPV	44%			92%		
NPV	72.3%			50%		
AUC ROC	0.833 (0.713–0.953)			0.749 (0.608–0.891)		
	*p* = 0.01			*p* = 0.002		

Abbreviations: AAP, American Academy of Periodontology; AUC ROC, area under the curve of the receiver operating characteristic curve; CDC, Center for Disease Control; EFP, European Federation of Periodontology; NPV, negative predictive value; PPV, positive predictive value. Sensitivity, specificity, PPV, NPV and AUC ROC are referred to for the diagnosis of atheroma plaque as the reference standard.

**Table 3:** Bivariate correlations between periodontal disease classifications and the variables analyzed.

Variable	CDC/AAP 2012	EFP/AAP 2018
Age (years old)	**r 0.283 *p* = 0.005**	**r 0.225 *p* = 0.028**
Gender	r 0.13.6 *p* = 0.183	r 0.030 *p* = 0.772
BMI (kg/m^2^)	**r 0.213 *p* = 0.045**	**r 0.282 *p* = 0.007**
Glucose (mg/dL)	r 0.164 *p* = 0.107	r 0.168 *p* = 0.099
HbA1c (%)	**r 0.250 *p* = 0.013**	r 0.079 *p* = 0.440
Apo B (mg/dL)	r 0.038 *p* = 0.710	r -0.066 *p* = 0.520
Smoking habit	**r 0.375 *p* = 0.001**	**r 0.250 *p* = 0.024**
Atheroma plaque	**r 0.522 *p* < 0.001**	**r 0.369 *p* < 0.001**
CDC/AAP 2012		**r 0.671 *p* < 0.001**
EFP/AAP 2018	**r 0.671 *p* < 0.001**	

Abbreviations: AAP, American Academy of Periodontology; Apo B, apolipoprotein B; BMI, body mass index; CDC, Center for Disease Control; EFP, European Federation of Periodontology; HbA1c, glycated hemoglobin. Bold indicates statistically significant.

**Table 4:** Logistic regression considering the presence of atheroma plaque as a dependent variable according to CDC/AAP (I) or EFP/AAP (II) criteria: A. subjects without diabetes; B. subjects with diabetes.

I.A.	Variable	B	Exp (B)	Significance
	Constant	−4.851	0.008	0.738
	Age (years old)	0.134	1.143	0.098
	Gender	1.545	4.690	0.241
	BMI (kg/m^2^)	0.049	1.051	0.627
	HbA1c (%)	−0.972	0.378	0.760
	Apo B (mg/dL)	−0.030	0.971	0.314
	**Smoking habit**	**1.916**	**6.794**	**0.034**
	CDC/AAP criteria	0.230	0.008	0.738
**I.B.**	**Variable**	**B**	**Exp (B)**	**Significance**
	Constant	−23.594	0.000	0.016
	**Age (years old)**	**0.225**	**1.252**	**0.009**
	Gender	2.695	14.799	0.112
	BMI (kg/m^2^)	0.041	1.042	0.741
	HbA1c (%)	0.129	1.138	0.820
	Apo B (mg/dL)	0.003	1.003	0.898
	Smoking habit	−1.178	0.308	0.249
	**CDC/AAP criteria**	**2.937**	**18.868**	**0.028**
**II.A.**	**Variable**	**B**	**Exp (B)**	**Significance**
	Constant	−5.408	0.004	0.712
	Age (years old)	0.135	1.145	0.099
	Gender	1.542	4.673	0.241
	BMI (kg/m^2^)	0.058	1.060	0.563
	HbA1c (%)	−1.154	0.315	0.704
	Apo B (mg/dL)	−0.028	0.972	0.320
	**Smoking habit**	**1.867**	**6.469**	**0.030**
	EFP/AAP criteria	0.510	1.666	0.366
**II.B.**	**Variable**	**B**	**Exp (B)**	**Significance**
	Constant	−13.959	0.000	0.058
	**Age (years old)**	**0.169**	**1.184**	**0.011**
	Gender	1.457	4.295	0.303
	BMI (kg/m^2^)	−0.107	0.898	0.414
	HbA1c (%)	−0.026	0.974	0.959
	Apo B (mg/dL)	0.018	1.018	0.444
	Smoking habit	−0.355	0.701	0.658
	EFP/AAP criteria	1.430	4.181	0.066

Abbreviations: AAP, American Academy of Periodontology; Apo B, apolipoprotein B; BMI, body mass index; CDC, Center for Disease Control; EFP, European Federation of Periodontology; HbA1c, glycated hemoglobin. Bold indicates statistically significant.

## Data Availability

The datasets generated during the current study are available from the corresponding author on reasonable request.
